# Disarming Pore-Forming Toxins with Biomimetic Nanosponges in Intraocular Infections

**DOI:** 10.1128/mSphere.00262-19

**Published:** 2019-05-15

**Authors:** Phillip S. Coburn, Frederick C. Miller, Austin L. LaGrow, Craig Land, Huzzatul Mursalin, Erin Livingston, Omar Amayem, Yijie Chen, Weiwei Gao, Liangfang Zhang, Michelle C. Callegan

**Affiliations:** a Department of Ophthalmology, University of Oklahoma Health Sciences Center, Oklahoma City, Oklahoma, USA; b Department of Cell Biology, University of Oklahoma Health Sciences Center, Oklahoma City, Oklahoma, USA; c Department of Family and Preventive Medicine, University of Oklahoma Health Sciences Center, Oklahoma City, Oklahoma, USA; d Department of Microbiology and Immunology, University of Oklahoma Health Sciences Center, Oklahoma City, Oklahoma, USA; e Dean McGee Eye Institute, Oklahoma City, Oklahoma, USA; f Department of NanoEngineering and Moores Cancer Centre, University of California, San Diego, San Diego, California, USA; Food and Drug Administration

**Keywords:** antibiotic, endophthalmitis, eye, infection, nanoparticle

## Abstract

Intraocular infections are prevalent after traumatic injuries or after common ocular surgeries. Infections cause inflammation that can damage the retina and architecture of the eye, often resulting in poor visual outcomes. Severe cases may result in blindness or require enucleation of the eye. Treatments for intraocular infections include intravitreal antibiotics and corticosteroids or surgical vitrectomy in serious cases. The increase in multidrug-resistant infections calls for novel treatment options. In the present study, a biomimetic erythrocyte-derived nanosponge was tested for the ability to neutralize pore-forming toxins from the most frequent Gram-positive bacterial causes of intraocular infections (Staphylococcus aureus, Enterococcus faecalis, Streptococcus pneumoniae, and Bacillus cereus). Nanosponge pretreatment of supernatants reduced hemolytic activity *in vitro.* In a murine sterile endophthalmitis model, nanosponge pretreatment of injected supernatants resulted in greater retinal function and less ocular pathology compared to that in eyes injected with untreated supernatants from all pathogens except methicillin-resistant S. aureus. In a murine bacterial endophthalmitis model, treatment with gatifloxacin and gatifloxacin-nanosponges reduced intraocular bacterial burdens, except in the case of methicillin-sensitive S. aureus. For all pathogens, eyes in both treatment groups showed decreased ocular pathology and inflammation. Overall, reductions in retinal function loss afforded by gatifloxacin-nanosponge treatment were significant for E. faecalis, S. pneumoniae, and methicillin-resistant S. aureus but not for B. cereus and methicillin-sensitive S. aureus. These results suggest that clinical improvements in intraocular infections following nanosponge treatment were dependent on the complexity and types of toxins produced. Nanosponges might serve as an adjunctive therapy for the treatment of ocular infections.

**IMPORTANCE** Endophthalmitis is a blinding consequence of bacterial invasion of the interior of the eye. Because of increases in the numbers of ocular surgeries and intraocular injections, the incidence of endophthalmitis is steadily increasing. Staphylococcus aureus, Enterococcus faecalis, Streptococcus pneumoniae, and Bacillus cereus are leading causes of infection following ocular procedures and trauma and are increasingly more difficult to treat due to multidrug resistance. Each of these pathogens produces pore-forming toxins that contribute to the pathogenesis of endophthalmitis. Treatment of these infections with antibiotics alone is insufficient to prevent damage to the retina and vision loss. Therefore, novel therapeutics are needed that include agents that neutralize bacterial pore-forming toxins. Here, we demonstrate that biomimetic nanosponges neutralize pore-forming toxins from these ocular pathogens and aid in preserving retinal function. Nanosponges may represent a new form of adjunct antitoxin therapy for serious potentially blinding intraocular infections.

## Abstract (precis)

Endophthalmitis is a blinding consequence of bacterial invasion of the interior of the eye. Because of increases in the numbers of ocular surgeries and intraocular injections, the incidence of endophthalmitis is steadily increasing. Staphylococcus aureus, Enterococcus faecalis, Streptococcus pneumoniae, and Bacillus cereus are leading causes of infection following ocular procedures and trauma and are increasingly more difficult to treat due to multidrug resistance. Each of these pathogens produces pore-forming toxins that contribute to the pathogenesis of endophthalmitis. Treatment of these infections with antibiotics alone is insufficient to prevent damage to the retina and vision loss. Therefore, novel therapeutics are needed that include agents that neutralize bacterial pore-forming toxins. Here, we demonstrate that biomimetic nanosponges neutralize pore-forming toxins from these ocular pathogens and aid in preserving retinal function. Nanosponges may represent a new form of adjunct antitoxin therapy for serious potentially blinding intraocular infections.

## INTRODUCTION

Bacteria are the primary cause of all blinding ocular infections that result in some degree of permanent disability. Among the bacterial etiological agents of ocular infections, the Gram-positive pathogens Bacillus cereus, Enterococcus faecalis, Staphylococcus aureus, and Streptococcus pneumoniae cause the vast majority of cases and rank among the most significant contributors to devastating visual outcomes (1–4). These four pathogens produce a myriad of toxins that contribute to the pathogenesis of ocular infections (1–4). Endophthalmitis, an infection involving the interior of the eye, is typically caused by bacteria which enter the posterior segment of the eye, replicate, produce toxins, and incite a robust inflammatory response (1–4). These events collectively damage essential and nonregenerative tissues which are responsible for proper vision. If not properly and promptly mitigated, these infections can result in loss of vision, blindness, or loss of the eye itself. Current treatment modalities for intraocular infections include the topical and intraocular administration of antibiotics and anti-inflammatory agents and, in the most severe cases, surgical vitrectomy (1–4). Despite this, outcomes can be poor, and patients can be left with count fingers vision to complete blindness. Furthermore, an increase in the recovery of antibiotic-resistant isolates from these infections (5) calls for alternative strategies to treat blinding intraocular infections.

Bacterial toxin production contributes to the severity and poor outcomes of ocular infections but is not addressed in current treatment regimens. We and others reported on the importance of bacterial toxins in endophthalmitis. B. cereus (6–8), E. faecalis (9–11), and S. aureus (12–14) strains deficient in the production of either single toxins or multiple toxins (quorum sensing deficient) were significantly attenuated in their ability to damage the retina, reduce retinal function, and incite robust inflammation compared to their wild-type parental strains. Immunization against the S. pneumoniae pneumolysin (15) was also beneficial in treating endophthalmitis (16, 17). While current treatment strategies can kill bacteria, secreted toxins remain in the infected tissue. Mitigating bacterial toxin activities might offer a therapeutic benefit in bacterial endophthalmitis.

We recently published the use of the biomimetic nanosponge in neutralizing enterococcal cytolysin activity in the eye, protecting mouse retinas from damage and loss of function (18). Nanosponges are nanoparticles surrounded by erythrocyte membranes which serve as a decoy for pore-forming toxins (PFTs) that would otherwise bind to and disrupt cell membranes. Nanosponges have been effective in arresting PFT activities in systemic S. aureus alpha-toxin toxemia and subcutaneous methicillin-resistant S. aureus (MRSA) infection. Nanosponges effectively protected mice from developing staphylococcal alpha-toxin-induced skin lesions and markedly reduced mortality rates after systemic injection of a lethal dose of alpha-toxin (19). Nanosponges also effectively reduced group A streptococcal streptolysin O-induced cell death (20, 21). The broad-spectrum antitoxin activity of human nanosponges has recently been reported (22).

The four Gram-positive genera noted above produce one or more PFTs which are significant contributors to the pathogenesis of ocular infections (6–17, 23–26). The current study expands our previous studies on the utility of nanosponges in protecting the eye from the damaging effects of bacterial PFTs and examines whether human nanosponges can be combined with an antibiotic (gatifloxacin) to treat bacterial endophthalmitis in mice. Here, we showed that rabbit nanosponges were able to effectively reduce the hemolytic activity of PFTs against erythrocytes *in vitro* and toxic activities during sterile endophthalmitis *in vivo*. Human nanosponges alone improved retinal function retention following intravitreal infection with E. faecalis and S. pneumoniae but not after infection with B. cereus or methicillin-sensitive S. aureus (MSSA). In conjunction with gatifloxacin, human nanosponges significantly improved retinal function retention over gatifloxacin alone following infection with MRSA. These results suggest that nanosponge effectiveness in preserving retinal function and architecture is dependent on the complexity and types of toxins produced and that nanosponges might improve the outcome of ocular infections without interfering with antibiotic effectiveness or inciting an inflammatory response. These results lay the foundation for further study of nanosponges as an adjunctive therapy for the treatment of ocular infections.

(This work was presented in part at the 2018 Association for Research in Vision and Ophthalmology meeting in Honolulu, HI.)

## RESULTS

### Rabbit nanosponges reduced PFT-mediated hemolysis *in vitro*.

**FIG 1:**
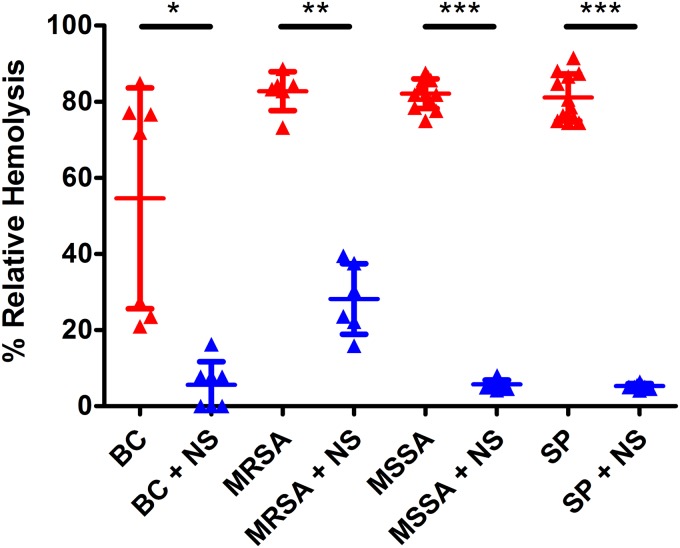
Rabbit nanosponges reduced hemolytic activity *in vitro*. Undiluted filter-sterilized supernatants were obtained from 18 h cultures of B. cereus strain ATCC 14579 (BC), methicillin-resistant S. aureus ocular isolate 180 (MRSA), methicillin-sensitive S. aureus strain 8325-4 (MSSA), and S. pneumoniae strain TIGR4 (SP), mixed 1:1 with 8 mg/ml rabbit nanosponges (NS) or PBS only, and allowed to incubate at 37°C for 30 min. Rabbit NS were removed by centrifugation and hemolytic activity was assessed. Values represent the means ± the standard deviations from three independent experiments. *, *P* = 0.0006; **, *P* = 0.005; ***, *P* < 0.0001 versus untreated controls.

We published that rabbit nanosponges significantly reduced the activity of the E. faecalis cytolysin by 68% after 30 min *in vitro* (18). To test the ability of rabbit nanosponges to neutralize PFTs from each of the other ocular bacterial pathogens, hemolysis assays were performed on culture supernatants using rabbit red blood cells. Dramatic reductions in the hemolytic activities of culture supernatants of all pathogens were observed after incubation with equal volumes of 8 mg/ml rabbit nanosponges for 30 min (Fig. 1). This concentration of rabbit nanosponges and incubation time yielded maximum reductions in hemolytic activity. Rabbit nanosponges reduced the hemolytic activity of B. cereus supernatant by 90% (*P* = 0.0006), MRSA supernatant by 66% (*P* = 0.005), MSSA supernatant by 93% (*P* < 0.0001), and S. pneumoniae supernatant also by 93% (*P* < 0.0001). These data indicate that regardless of the bacterial species, rabbit nanosponges are capable of significantly reducing the activity of a broad range of PFTs *in vitro*.

### Rabbit nanosponge neutralization of PFTs improved retinal function retention in a sterile endophthalmitis model.

**FIG 2:**
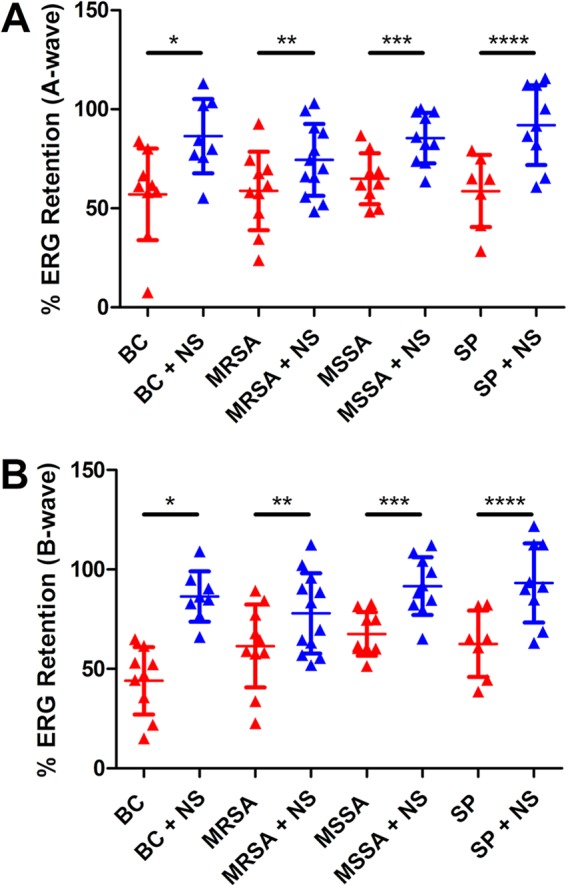
Rabbit nanosponge neutralization of PFTs improved retinal function retention in a sterile endophthalmitis model. Right eyes of C57BL/6J mice were injected with either 0.5 μl of rabbit nanosponge (NS)-treated bacterial supernatant or untreated bacterial supernatant from either B. cereus (BC), methicillin-resistant S. aureus (MRSA), methicillin-sensitive S. aureus (MSSA), or S. pneumoniae (SP). Retinal function was assessed by electroretinography 24 h postinjection. Eyes injected with either rabbit NS-treated BC, MSSA, or SP supernatants had significantly higher A-wave (*, *P* = 0.0274; ***, *P* = 0.0041; ****, *P* = 0.0052) (A) and B-wave (*, *P* < 0.0001; ***, *P* = 0.001; ****, *P* = 0.0052) (B) retention versus that in untreated controls. Eyes injected with rabbit NS-treated MRSA supernatant did not exhibit a significantly increased A-wave (**, *P* = 0.1213) (A) or B-wave (**, *P* = 0.1765) (B) retention relative to that in untreated controls. Values represent the means ± the standard deviations from at least 7. Two independent experiments were performed.

We published that rabbit nanosponge pretreatment of E. faecalis culture supernatants prior to intravitreal injection resulted in a 270% to 439% increase in retinal function retention compared to that in eyes injected with supernatants that were not treated with nanosponges (18). To test whether the rabbit nanosponges were capable of neutralizing PFTs and improving visual outcomes *in vivo*, scotopic electroretinography (ERG) was performed on mice 24 h after their eyes were injected with either 1 μl of supernatants diluted 1:2 in phosphate-buffered saline (PBS) or supernatants treated with 8 mg/ml of rabbit nanosponges for 30 min. With the exception of MRSA, the mean A-wave amplitude retentions were significantly greater in the rabbit nanosponge-treated groups than in the untreated groups. The mean A-wave retention of the treated group was 52% greater for B. cereus (*P* = 0.0274), 32% greater for MSSA (*P* = 0.0041), and 57% greater for S. pneumoniae (*P* = 0.0052) than for the respective untreated groups (Fig. 2A). The mean A-wave retention was 27% higher in the treated group relative to the untreated group for MRSA, but this difference was not significant (*P* = 0.1213) (Fig. 2A). The mean B-wave retention of the treated groups was 96% greater for B. cereus (*P* < 0.0001), 36% greater for MSSA (*P* = 0.001), and 49% greater for S. pneumoniae (*P* = 0.0052) than for the untreated groups (Fig. 2B). For the MRSA isolate, the mean B-wave retention was 27% higher in the treated group than in the untreated, but this difference was also not significant (*P* = 0.1765) (Fig. 2B). With exception of supernatant from the MRSA isolate, these results indicated that PFTs were sufficiently neutralized by the rabbit nanosponges to protect retinal function retention *in vivo*.

### Rabbit nanosponges neutralized PFTs and protected the retina from PFT-mediated damage in a sterile endophthalmitis model.

**FIG 3:**
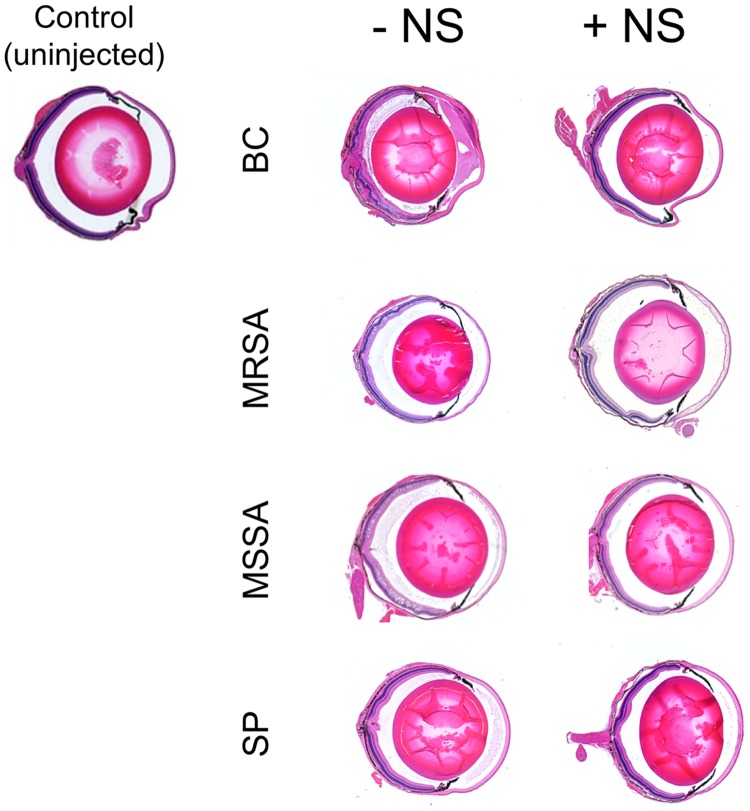
Rabbit nanosponges neutralized PFTs and protected the retina from PFT-mediated damage in a sterile endophthalmitis model. Right eyes were injected with either 0.5 μl of rabbit nanosponge (NS)-treated B. cereus (BC), methicillin-resistant S. aureus (MRSA), methicillin-sensitive S. aureus (MSSA), S. pneumoniae (SP) supernatant, or untreated supernatant. Eyes were then harvested 24 h later and processed for hematoxylin and eosin staining. Images are representative of at least 3 eyes from 2 independent experiments. Control uninjected mouse eyes did not exhibit inflammatory infiltrate in either the anterior or posterior chambers, and the corneas and retinal layers appeared normal. Eyes injected with BC, MRSA, MSSA, and SP typically showed retinal and corneal edema and cellular infiltrate and fibrin deposition in the anterior and posterior segments. In eyes injected with rabbit NS-treated BC, MRSA, MSSA, and SP supernatant showed less anterior and posterior chamber infiltrate, less retinal edema, intact retinal layers, and normal corneas.

Untreated eyes and eyes injected with supernatants from each of the strains that were not treated or treated with rabbit nanosponges were analyzed by histology (Fig. 3). Control uninjected mice had normal corneas, intact retinal layers, and no inflammation. Eyes injected with B. cereus supernatant had retinal and corneal edema, cellular infiltrate in the posterior segment, fibrinous exudates in both the anterior and posterior chambers, and some retinal architecture disruption. Eyes injected with rabbit nanosponge-treated B. cereus supernatant had comparatively less retinal edema and anterior and posterior chamber infiltrate than eyes injected with untreated supernatant. These eyes also had normal-appearing corneas. In eyes injected with either MRSA or MSSA supernatant, cellular infiltrate and fibrin deposition was visible in the vitreous as well as anterior chamber edema, and the retinal layers were also somewhat disrupted (Fig. 3). Relative to the eyes injected with untreated supernatants, less infiltrate and fibrin deposition and normal retinal layering were observed after injections of rabbit nanosponge-treated MRSA or MSSA supernatant (Fig. 3). Eyes injected with S. pneumoniae supernatant exhibited retinal edema, cellular infiltration, and fibrin deposition that were all comparatively reduced in eyes injected with rabbit nanosponge-treated S. pneumoniae supernatant (Fig. 3). These results indicated that rabbit nanosponge depletion of PFT activity from supernatants from all four pathogens prior to injection into the vitreous resulted in decreased inflammation and preserved retinal architecture.

### Human nanosponge effectiveness in preserving retinal function and architecture is dependent on the complexity and types of toxins produced.

**FIG 4:**
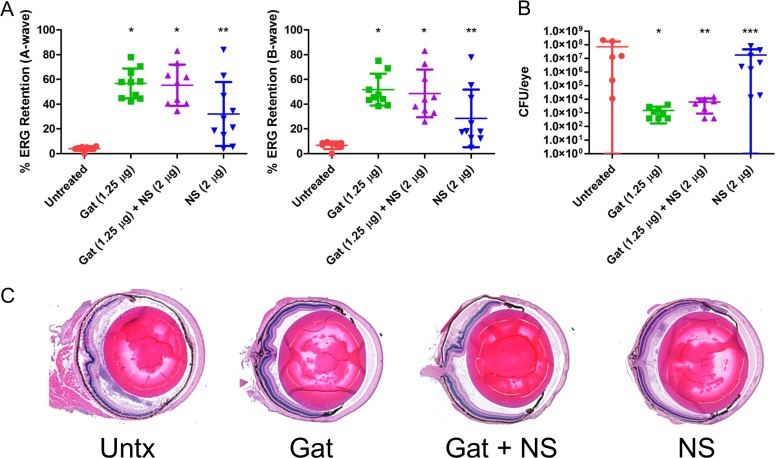
Human nanosponges and human nanosponges plus gatifloxacin increased retinal function retention and protected retinal architecture following E. faecalis infection in a murine model of endophthalmitis. Right eyes of mice were infected with 100 CFU of E. faecalis. At 6 h postinfection, E. faecalis-infected right eyes were intravitreally injected with 0.5 μl PBS only (untreated control), 0.5 μl PBS containing 1.25 μg gatifloxacin (Gat), 0.5 μl PBS containing 1.25 μg gatifloxacin and 2 μg of human nanosponges (Gat+NS), or 0.5 μl PBS containing 2 μg of human nanosponges (NS). (A) Retinal function was assessed by electroretinography 24 h postinfection. Values represent means ± standard deviations (SDs) from at least 6 eyes per group in two independent experiments (A-wave: *, *P* < 0.0001; **, *P* = 0.0014; B-wave: *, *P* < 0.0001; **, *P* = 0.0021 versus untreated controls). (B) Eyes were harvested from mice and E. faecalis CFU/eye was determined. Values represent means ± SDs from at least 6 eyes per group in two independent experiments (*, *P* = 0.0007; **, *P* = 0.0013; ***, *P* = 0.5728 versus untreated controls). (C) Histological analysis of eyes infected with E. faecalis revealed retinal and corneal edema and cellular infiltration and fibrin deposition in both the anterior and posterior chambers; partial disruption of retinal layers was also apparent. In eyes treated with Gat and Gat+NS, retinal dissolution and edema, cellular infiltration, and fibrin deposition were reduced relative to that in untreated eyes. Eyes treated with NS only showed intact retinal layers, but cellular infiltrates and fibrin were not reduced in the anterior and posterior segments relative to that in untreated eyes.

The cytolysin is a significant contributor to E. faecalis virulence in endophthalmitis and is primarily responsible for retinal toxicity (9, 10). It is also the only PFT produced by E. faecalis, and human cells are sensitive to its hemolytic and cytotoxic effects (27, 28). We hypothesized that human nanosponges might be more effective against cytolytic E. faecalis than against the other Gram-positive endophthalmitis pathogens that produce a multitude of non-PFTs in addition to PFTs. We first tested the ability of human nanosponges to neutralize the cytolysin and attenuate E. faecalis infection in our mouse model. We published that intravitreal injection of rabbit nanosponges 6 h following infection with a cytolytic E. faecalis strain resulted in significantly greater A-wave and B-wave retention relative to that in untreated controls (18). To further test this with human nanosponges, and to determine whether the addition of gatifloxacin would enhance the effect of the human nanosponges in our model, mouse eyes were infected with 100 CFU of a cytolytic E. faecalis strain, and at 6 h postinfection, mice were intravitreally injected with PBS only (untreated control), PBS with 1.25 μg gatifloxacin, PBS with 1.25 μg gatifloxacin plus 2 μg human nanosponges, or PBS with 2 μg human nanosponges alone. At 24 h postinfection, scotopic ERG was performed to assess retinal function retention, and eyes were harvested for either histological analysis to examine ocular pathology or for CFU determination. Eyes infected with cytolytic E. faecalis demonstrated a mean A-wave retention of 4.1% and B-wave retention of 6.8% (Fig. 4A). In eyes treated with gatifloxacin, the mean A-wave retention was 56.8% (*P* < 0.0001) and B-wave retention was 51.8% (*P* < 0.0001) (Fig. 4A), significantly higher retentions than in the untreated group. The gatifloxacin-treated group had a mean concentration of 1.5 × 10^3^ CFU per eye versus 7.1 × 10^7^ CFU per eye for the untreated group (*P* = 0.0007) (Fig. 4B). The addition of human nanosponges to gatifloxacin also resulted in significantly greater A-wave (55.4%, *P* < 0.0001) and B-wave (48.7%, *P* < 0.0001) retention compared to that in the untreated controls (Fig. 4A). The mean E. faecalis concentration in the gatifloxacin plus nanosponge group was 6.2 × 10^3^ CFU/eye, significantly lower than in the untreated group (*P* = 0.0013) at 24 h postinfection (Fig. 4B). However, this was not significantly different from eyes in the treatment with gatifloxacin alone group (*P* = 0.0878), suggesting that human nanosponges did not interfere with the activity of gatifloxacin *in vivo*. The addition of human nanosponges did not augment the activity of gatifloxacin in lowering the bacterial burden and therefore improving retinal function retention (*P* = 0.6038 for A-wave and *P* = 0.4002 for B-wave). Treatment with human nanosponges alone also resulted in an A-wave retention of 32.1% (*P* = 0.0014) and B-wave retention of 28.5% (*P* = 0.0021) (Fig. 4A) but did not significantly alter the growth of E. faecalis, as the mean CFU per eye was 1.7 × 10^7^ (*P* = 0.5728 versus untreated controls) (Fig. 4B). The combination of human nanosponges and gatifloxacin resulted in significantly higher A-wave (*P* = 0.0350) and B-wave (*P* = 0.0350) retentions than treatment with human nanosponges alone. Histological analysis of infected untreated eyes revealed retinal and corneal edema, cellular infiltration, and fibrin deposition in both the anterior and posterior chambers, and partial dissolution of the retinal layers (Fig. 4C). Eyes treated with gatifloxacin showed less retinal dissolution than untreated eyes, but corneal edema was still present. Cellular infiltration and fibrin deposition were markedly reduced in both chambers (Fig. 4C) relative to that in untreated eyes. Eyes treated with gatifloxacin plus human nanosponges were similar in appearance to eyes treated only with gatifloxacin (Fig. 4C). Eyes treated only with human nanosponges had intact retinal layers, but considerable cellular infiltrates and fibrin were present in the anterior and posterior segments (Fig. 4C). These results indicated that human nanosponges alone or in combination with gatifloxacin improved retinal function and reduced ocular pathology following infection with cytolysin-producing E. faecalis. Furthermore, human nanosponges did not affect the *in vivo* activity of gatifloxacin toward E. faecalis.

**FIG 5:**
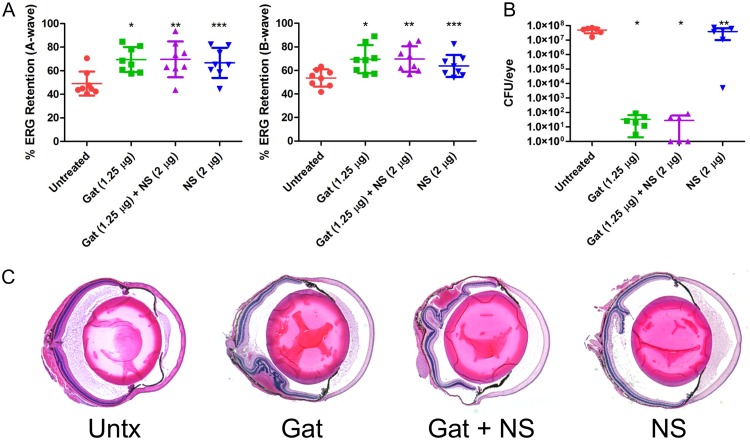
Human nanosponges increased retinal function retention and preserved retinal architecture but did not influence growth following S. pneumoniae infection in a murine model of endophthalmitis. Right eyes of mice were infected with 100 CFU of S. pneumoniae. At 6 h postinfection, S. pneumoniae-infected right eyes were intravitreally injected with 0.5 μl PBS only (untreated control), 0.5 μl PBS containing 1.25 μg gatifloxacin (Gat), 0.5 μl PBS containing 1.25 μg gatifloxacin and 2 μg of human nanosponges (Gat+NS), or 0.5 μl PBS containing 2 μg of human nanosponges (NS). (A) Retinal function was assessed by electroretinography 24 h postinfection. Values represent means ± SDs from at least 6 eyes per group in two independent experiments (A-wave: *, *P* = 0.0047; **, *P* = 0.0156; ***, *P* = 0.0148; B-wave: *, *P* = 0.0070; **, *P* = 0.0047; ***, *P* = 0.0379 versus untreated controls). (B) Eyes were harvested from mice and S. pneumoniae CFU/eye was determined. Values represent means ± SDs from at least 6 eyes per group in two independent experiments (*, *P* = 0.0022; **, *P* = 0.5887 versus untreated controls). (C) Histopathological analysis of untreated and treated eyes revealed an edematous retina and cornea and cellular infiltration and fibrinous deposition in both anterior and posterior segments in untreated eyes. Eyes treated with either Gat or NS showed less retinal edema, cellular infiltration, and fibrin deposition in both chambers compared to that in untreated eyes. Eyes treated with both Gat and NS appeared essentially normal.

Similar to E. faecalis, S. pneumoniae produces a single PFT, pneumolysin, which contributes to the pathogenesis of S. pneumoniae endophthalmitis (15–17). Vaccination of mice with a detoxified pneumolysin elicited neutralizing antipneumolysin antibodies that reduced ocular damage following intravitreal injection of S. pneumoniae (17). These results suggest a potential therapeutic benefit of neutralizing pneumolysin to improve the outcome of endophthalmitis. To test this hypothesis, the right eyes of mice were intravitreally injected with 100 CFU of S. pneumoniae and treated with gatifloxacin, gatifloxacin plus human nanosponges, or human nanosponges alone at 6 h postinfection as described above. At 24 h postinfection, untreated mice had a mean A-wave retention of 49.1% and B-wave retention of 53.6% (Fig. 5A). Mice treated with gatifloxacin exhibited a mean A-wave of 69.4% (*P* = 0.0047) and B-wave of 69.5% (*P* = 0.0070), significantly higher than in the untreated controls. Human nanosponge treatment alone resulted in a similarly higher mean A-wave of 66.7% (*P* = 0.0148) and B-wave of 63.8% (*P* = 0.0379) (Fig. 5A) than in untreated control mice. However, human nanosponge treatment was not significantly different from treatment with gatifloxacin alone (*P* = 0.8785 for A- and B-waves). Moreover, treatment with gatifloxacin and human nanosponges did not afford increased protection over gatifloxacin or human nanosponges alone. The mean A-wave retention in mice treated with both was 69.6% (*P* = 0.0156) and B-wave retention was 69.7% (*P* = 0.0047) versus untreated controls (Fig. 5A). The mean concentration of S. pneumoniae in eyes treated with gatifloxacin was 33 CFU/eye as opposed to 4.96× 10^7^ CFU/eye in untreated mouse eyes (*P* = 0.0022) (Fig. 5B). The mean concentration of S. pneumoniae in eyes treated with both gatifloxacin and human nanosponges was 28 CFU/eye (*P* = 0.0022), whereas eyes that were treated with human nanosponges alone had comparable numbers of bacteria to those in the untreated controls (3.8 × 10^7^ CFU/eye, *P* = 0.5887) (Fig. 5B). Histopathological differences between untreated and treated eyes were obvious (Fig. 5C). Untreated eyes had edematous retinas and corneas and cellular infiltration and fibrinous deposition in both anterior and posterior segments. Eyes treated with either gatifloxacin or human nanosponges showed less retinal edema, cellular infiltration, and fibrin deposition in both chambers relative to untreated eyes. Eyes treated with both gatifloxacin and human nanosponges (Fig. 5C) appeared equivalent to normal uninfected eyes (Fig. 3). Taken together, these results showed that treatment with human nanosponges or gatifloxacin resulted in similar levels of retinal function preservation. However, human nanosponges did not provide an additive effect to gatifloxacin. These data also suggest that pneumolysin neutralization is as important as reducing S. pneumoniae bacterial numbers in improving retinal function.

**FIG 6:**
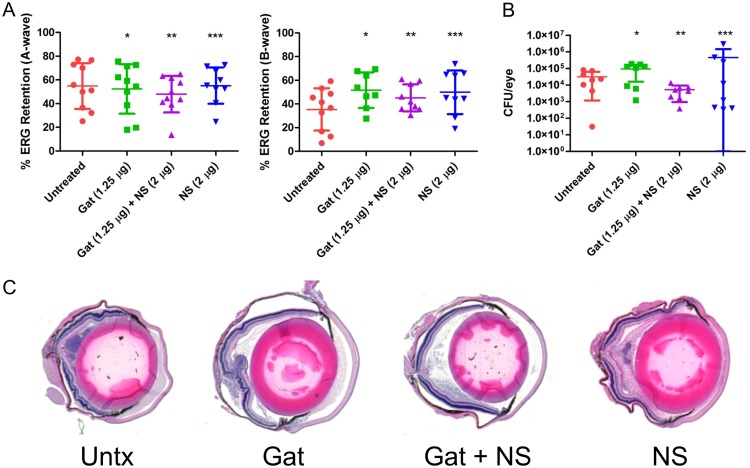
Human nanosponges did not influence retinal function retention, retinal architecture, and growth following MSSA infection in a murine model of endophthalmitis. Right eyes of mice were infected with 5,000 CFU of MSSA. At 6 h postinfection, MSSA-infected right eyes were intravitreally injected with 0.5 μl PBS only (untreated control), 0.5 μl PBS containing 1.25 μg gatifloxacin (Gat), 0.5 μl PBS containing 1.25 μg gatifloxacin and 2 μg of human nanosponges (Gat+NS), or 0.5 μl PBS containing 2 μg of human nanosponges (NS). (A) Retinal function was assessed by electroretinography 24 h postinfection. Values represent means ± SDs from at least 6 eyes per group in two independent experiments (A-wave: *, *P* = 0.7959; **, *P* = 0.4813; ***, *P* = 0.9682; B-wave: *, *P* = 0.0831; **, *P* = 0.3154; ***, *P* = 0.1333 versus untreated controls). (B) Eyes were harvested from mice and MSSA CFU/eye was determined. Values represent means ± SDs from at least 6 eyes per group in two independent experiments (*, *P* = 0.1949; **, *P* = 0.0585; ***, *P* = 0.9591 versus untreated controls). (C) Histopathology revealed severe inflammation in untreated eyes, with cellular infiltration and fibrinous deposition in both chambers and severe retinal and corneal edema. The Gat- and Gat+NS-treated groups showed a decrease in inflammation in terms of decreased infiltrate and fibrin in both chambers compared to that in untreated eyes. The NS-treated group appeared similar to the untreated group. In all groups, the layers of the retina remained relatively intact.

Whereas both E. faecalis and S. pneumoniae produce only a single PFT, each of which were efficiently disarmed by nanosponges, S. aureus presents a more complicated therapeutic challenge due to the production of a broad array of both PFTs and non-PFTs. S. aureus possesses an arsenal of PFTs, including alpha- and beta-toxins, six bicomponent leukocidins, and phenol-soluble modulins (29, 30). In our sterile endophthalmitis model, pretreatment of MSSA supernatants with rabbit nanosponges reduced PFT activity and improved retinal function (Fig. 1 and 2). To determine whether human nanosponges would be effective in our live infection endophthalmitis model, mice were intravitreally injected with 5,000 CFU of MSSA, followed by treatment with gatifloxacin, gatifloxacin plus human nanosponges, or human nanosponges alone 6 h postinfection as described above. After 24 h, untreated mouse eyes showed A- and B-wave retentions of 54.8% and 35.3%, respectively (Fig. 6A). Surprisingly, gatifloxacin treatment did not result in protection of retinal function, as A- and B-wave retentions were similarly reduced to 52.3% (*P* = 0.7959) and 51.7% (*P* = 0.0831), respectively, relative to that in untreated control mice (Fig. 6A). Human nanosponges alone, or human nanosponges in conjunction with gatifloxacin, also failed to protect retinal function, as retention and outcomes were similar to those of untreated mice. Gatifloxacin plus human nanosponges resulted in an A-wave retention of 48.0% (*P* = 0.4813) and B-wave retention of 45.1% (*P* = 0.3154) (Fig. 6A). Nanosponges alone resulted in an A-wave retention of 55.2% (*P* = 0.9682) and B-wave retention of 49.9% (*P* = 0.1333) (Fig. 6A). Interestingly, treatment with gatifloxacin, regardless of the presence of human nanosponges, did not affect the concentration of bacteria in the eyes, as there was no significant difference in CFU/eye across the untreated and treatment groups. The mean CFU/eye was 3.2 × 10^4^ for the untreated group, 9.3 × 10^4^ for the gatifloxacin-treated group, 5.1 × 10^3^ for the gatifloxacin plus human nanosponges-treated group, and 4.5 × 10^5^ for the human nanosponges only group (Fig. 6B). Examination of tissue sections revealed severe inflammation in both untreated and human nanosponge-treated mice (Fig. 6C). Both groups showed cellular infiltration and fibrinous deposition in both chambers and severe retinal and corneal edema. While both gatifloxacin and human nanosponges, alone or in combination, did not alter MSSA concentrations in the eye, the gatifloxacin-treated eyes were less inflamed than untreated eyes in terms of decreased infiltrate and fibrin in both chambers (Fig. 6C). In all groups, the layers of the retina remained relatively intact. These results show that human nanosponges were ineffective at improving retinal function following MSSA infection in our mouse model and that while gatifloxacin reduced inflammation alone and when combined with human nanosponges, human nanosponges were unable to sufficiently detoxify MSSA *in vivo* and improve the outcome of infection.

**FIG 7:**
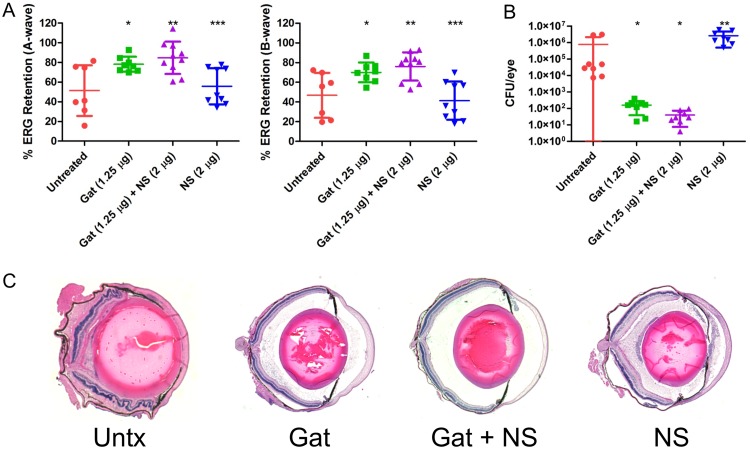
Human nanosponges augmented gatifloxacin in increasing retinal function retention and preserving retinal architecture but did not influence growth following MRSA infection in a murine model of endophthalmitis. Right eyes of mice were infected with 5,000 CFU of MRSA. At 6 h postinfection, MRSA-infected right eyes were intravitreally injected with 0.5 μl PBS only (untreated control), 0.5 μl PBS containing 1.25 μg gatifloxacin (Gat), 0.5 μl PBS containing 1.25 μg gatifloxacin and 2 μg of human nanosponges (Gat+NS), or 0.5 μl PBS containing 2 μg of human nanosponges (NS). (A) Retinal function was assessed by electroretinography 24 h postinfection. Values represent means ± SDs from at least 6 eyes per group in two independent experiments (A-wave: *, *P* = 0.0721; **, *P* = 0.0097; ***, *P* = 0.7577; B-wave: *, *P* = 0.0541; **, *P* = 0.0136; ***, *P* = 0.6806 versus untreated controls). (B) Eyes were harvested from mice and MRSA CFU/eye was determined. Values represent means ± SDs from at least 6 eyes per group in two independent experiments (*, *P* = 0.0002; **, *P* = 0.0207 versus untreated controls). (C) Histopathological analysis showed severe retinal and corneal swelling, alterations in the architecture of the retina, and cellular infiltration of the posterior segment in untreated eyes. Gat-treated eyes showed reduced retinal and corneal edema and decreased cellular infiltration, and retinal layers appeared normal compared to those in untreated eyes. Eyes treated with Gat+NS were similar to uninfected normal controls. NS-treated eyes appeared to have reduced inflammation relative to untreated eyes; however, cellular infiltrate and fibrinous deposition was still observed in both the anterior and posterior segments.

Given that MRSA is increasingly isolated from cases of endophthalmitis (31–33), we also sought to test the effectiveness of the human nanosponges toward an MRSA endophthalmitis isolate. Infection of mouse eyes with 5,000 CFU of MRSA resulted in 51.4% A-wave retention and 46.8% B-wave retention after 24 h (Fig. 7A). Treatment with gatifloxacin resulted in an A-wave retention of 78.1% and B-wave retention of 70.1%. However, this was not a statistically significant difference from those in untreated eyes (A-wave, *P* = 0.0721; B- wave, *P* = 0.0541) (Fig. 7A). Mouse eyes treated with gatifloxacin plus human nanosponges had significantly improved A-wave retention (84.7%, *P* = 0.0097) and B-wave retention (76.1%, *P* = 0.0136), suggesting that inclusion of human nanosponges enhanced the effect of gatifloxacin in improving retinal function (Fig. 7A). However, nanosponges alone did not mitigate retina functional loss. Human nanosponge-treated eyes had a mean A-wave retention of 55.8% (*P* = 0.7577) and B-wave retention of 41.3% (*P* = 0.6806) at 24 h postinfection (Fig. 7A). Gatifloxacin treatment resulted in an MRSA concentration of 159 CFU/eye compared to 7.56 × 10^5^ CFU/eye in untreated mouse eyes (*P* = 0.0002) (Fig. 7B). In mouse eyes treated with gatifloxacin plus human nanosponges, the MRSA concentration decreased to a mean of 40 CFU/eye (*P* = 0.0002 versus untreated) (Fig. 7B). Interestingly, human nanosponges enhanced the antibacterial effect of gatifloxacin, as this represented a significant decrease over gatifloxacin alone (*P* = 0.0356). MRSA concentrations in human nanosponge-treated mouse eyes were slightly elevated compared to those in untreated eyes, with mean concentrations of 2.58 × 10^6^ CFU/eye (*P* = 0.0207). Similarly to infection with the MSSA strain, untreated infected eyes had severe retinal and corneal swelling, disruption of the normal layering of the retina, and cellular infiltration of the posterior segment (Fig. 7C). In eyes treated with gatifloxacin, retinal and corneal edema was markedly reduced, fewer infiltrating inflammatory cells were observed, and retinal layers appeared normal (Fig. 7C) compared to untreated eyes. Eyes treated with gatifloxacin plus human nanosponges appeared indistinguishable from control uninfected eyes (Fig. 7C). While inflammation was reduced in nanosponge-treated eyes relative to that in untreated eyes, inflammatory infiltrate and fibrinous deposition was still observed in both the anterior and posterior segments (Fig. 7c). These results indicate that human nanosponges enhanced the *in vivo* activity of gatifloxacin toward this MRSA isolate and thereby augmented gatifloxacin in increasing retinal function and reducing inflammation following MRSA infection.

**FIG 8:**
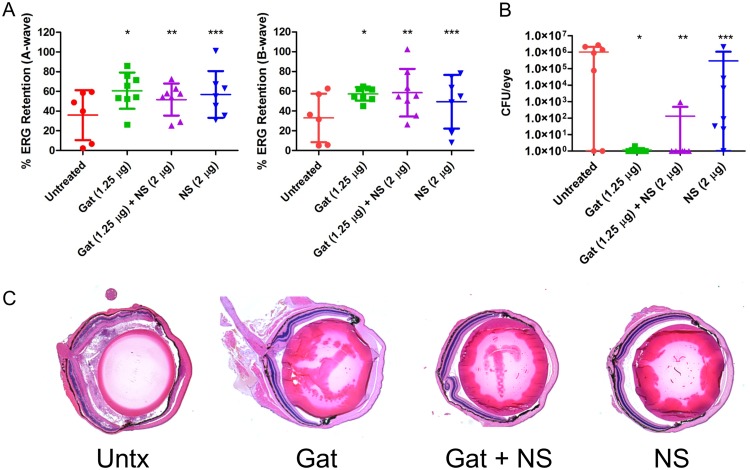
Human nanosponges did not influence retinal function retention and growth but preserved retinal architecture following B. cereus infection in a murine model of endophthalmitis. Right eyes of mice were infected with 100 CFU of B. cereus. At 6 h postinfection, B. cereus-infected right eyes were intravitreally injected with 0.5 μl PBS only (untreated control), 0.5 μl PBS containing 1.25 μg gatifloxacin (Gat), 0.5 μl PBS containing 1.25 μg gatifloxacin and 2 μg of human nanosponges (Gat+NS), or 0.5 μl PBS containing 2 μg of human nanosponges (NS). (A) Retinal function was assessed by electroretinography 12 h postinfection. Values represent means ± SDs from at least 6 eyes per group in two independent experiments (A-wave: *, *P* = 0.0813; **, *P* = 0.3450; ***, *P* = 0.2949; B-wave: *, *P* = 0.0813; **, *P* = 0.1812; ***, *P* = 0.2343 versus untreated controls). (B) Eyes were harvested from mice and B. cereus CFU/eye was determined. Values represent means ± SDs from at least 6 eyes per group in two independent experiments (*, *P* = 0.0407; **, *P* = 0.0236; ***, *P* = 0.1435 versus untreated control). (C) Histological examination of untreated B. cereus-infected eyes showed retinal and corneal edema, cellular infiltrates and fibrinous exudates in the anterior and posterior segments, and retinal layer disruption. In mouse eyes treated with Gat, Gat+NS, and NS alone, less anterior and posterior segment cellular infiltration and fibrin deposition were observed, retinal and corneal edema was reduced, and retinal layers were intact compared to untreated eyes.

B. cereus also produces a wide array of both PFTs and non-PFTs that together contribute to the rapid and virulent course of B. cereus endophthalmitis (6–8). Among the PFTs, cereolysin O (hemolysin I), hemolysin II, hemolysin III, and two hemolytic enterotoxins, Hbl and CytK, represent potential human nanosponge targets (34). We published that deletion of the genes encoding Hbl did not alter the course or severity of B. cereus endophthalmitis, suggesting that disarming Hbl would be insufficient to mitigate disease outcome (35). Nevertheless, red blood cell (RBC)-coated nanoparticles have a broad specificity for PFTs, and disarming multiple B. cereus PFTs might mitigate the highly virulent course of B. cereus endophthalmitis. To test this hypothesis, we infected mouse eyes with 100 CFU of B. cereus, and at 6 h postinfection, mice were treated with gatifloxacin, gatifloxacin plus human nanosponges, or human nanosponges alone as described above. At 12 h postinfection, mouse eyes infected with B. cereus demonstrated a mean A-wave retention of 35.9% and B-wave retention of 33.1% (Fig. 8A). The A-wave retention was 60.7% and B-wave retention was 57.4% for eyes treated with gatifloxacin, but this did not represent a statistically significant difference (*P* = 0.0813 for both A- and B-waves) compared to control untreated eyes. The addition of human nanosponges to gatifloxacin did not significantly improve A-wave (51.7%, *P* = 0.3450) or B-wave (58.5%, *P* = 0.1812) responses, nor did human nanosponges alone (A-wave of 56.9%, *P* = 0.2949; B-wave of 49.4%, *P* = 0.2343) (Fig. 8A). To assess whether B. cereus was sufficiently killed by gatifloxacin, bacterial CFU were determined from a subset of mice following ERG. The mean CFU from untreated eyes was 1.0 × 10^6^ CFU/eye, and treatment with gatifloxacin resulted in a concentration of <1 CFU/eye (*P* = 0.0407) (Fig. 8B). These results indicated that the lack of a significant effect on retinal function retention was not due to insufficient bacterial killing. Treatment with gatifloxacin and human nanosponges combined resulted in a concentration of 130 CFU/eye. The difference between the bacterial concentrations following treatment with gatifloxacin and gatifloxacin plus nanosponges was not significant (*P* = 0.7221), indicating that human nanosponges did not alter the *in vivo* activity of gatifloxacin toward B. cereus (Fig. 8B). Human nanosponges alone did not significantly alter the growth of B. cereus as the mean level of growth following treatment with nanosponges was 2.96 × 10^5^ CFU/eye (*P* = 0.1435 versus untreated control) (Fig. 8B). Histological examination of untreated, B. cereus-infected eyes showed severe and extensive retinal and corneal edema, cellular infiltrate and fibrinous exudates in both the anterior and posterior chambers, and disruption of the retinal layers (Fig. 8C). However, compared to untreated eyes, mouse eyes treated with gatifloxacin, gatifloxacin plus human nanosponges, or human nanosponges alone showed less anterior segment infiltrate and fibrin deposition and reduced retinal and corneal edema, and retinal layers were intact (Fig. 8C). These results indicated that while gatifloxacin or human nanosponges alone, or in combination, reduced ocular pathology, this treatment strategy did not significantly preserve retinal function. These results also showed that human nanosponges did not affect the *in vivo* activity of gatifloxacin toward B. cereus.

## DISCUSSION

The four most common Gram-positive etiological agents of endophthalmitis, E. faecalis, S. pneumoniae, S. aureus, and B. cereus, elaborate a myriad of PFTs that have been ascribed key roles in the course and severity of intraocular infections. The E. faecalis cytolysin exerted a direct toxic effect on retinal cells, disrupted retinal architecture, and played an important role in dictating the course and severity of disease (9, 10). Pneumolysin-mediated retinal damage was significantly reduced in rabbits immunized with a nontoxic derivative of pneumolysin compared to that in nonimmunized rabbits following intraocular S. pneumoniae infection (17). Secreted proteins from B. cereus and S. aureus were shown to damage the retina and incite an inflammatory response (36), and both S. aureus alpha- and beta-toxins were shown to play a role in retinal damage in a rabbit model of endophthalmitis (14). Inactivation of the S. aureus global toxin regulators Agr and Sar resulted in an attenuated course of infection and a decrease in damage to the retina (13). Similar results were achieved by inactivating the B. cereus global PFT regulator PlcR and the quorum-sensing regulator *fsr* of E. faecalis that regulates a secreted serine protease and a gelatinase (6, 11). These studies validated the importance of PFTs to the course and visual outcome of intraocular infection and suggest that neutralizing bacterial PFTs might improve the prognosis of intraocular infections. Strategies for treating bacterial endophthalmitis typically consist of the ophthalmologist’s choice of vancomycin, ceftazidime, amikacin, and/or expanded-spectrum fluoroquinolones, corticosteroids for anti-inflammation, and vitrectomy (37). While this therapeutic strategy is designed to sterilize the eye and reduce inflammation, bacterial toxin production during intraocular infection is not addressed.

The development and application of therapeutic biomimetic nanoparticles represents an important and exciting advance in the treatment of infectious diseases. Nanoparticles have been developed that target both bacterial toxins and the host inflammatory response. Parodi et al. developed an inflammation-targeting nanoparticle composed of a combination of membranes of macrophages, monocytes, and T lymphocytes that targeted activated human umbilical vein endothelial cells *in vitro* and effectively crossed endothelial barriers *in vivo* (38). Thamphiwatana et al. developed macrophage-based nanosponge that effectively bound and depleted the Escherichia coli endotoxin lipopolysaccharide (LPS) as well as the proinflammatory cytokines interleukin 6 (IL-6), tumor necrosis factor alpha (TNF-α), and gamma interferon (IFN-γ) *in vitro* (39). These macrophage-based nanosponges showed promise *in vivo* by functioning as a detoxification and anti-inflammatory particle. Administration of macrophage nanosponges protected mice following a lethal challenge of E. coli (39). Proinflammatory cytokine levels were reduced, as were bacterial counts in the blood and spleens. Nanoparticles have also been engineered that possess natural RBC membranes surrounding a biologically inert poly(lactic-co-glycolic acid (PLGA) core and are capable of intercepting and neutralizing a broad spectrum of bacterial PFTs (19–22). RBC-derived nanosponges effectively neutralized the S. aureus PFT alpha-toxin and prevented tissue damage in a mouse dermonecrotic lesion model (19). Nanosponges neutralized the Streptococcus pyogenes streptolysin O (SLO) toxin and reduced lysis of RBCs, keratinocyte and macrophage cell death, and disease severity in a necrotizing skin infection mouse model (21). Chen et al. recently reported the ability of RBC-derived nanosponges to neutralize an array of PFTs, including S. aureus alpha-toxin, S. pyogenes SLO, and Listeria monocytogenes listeriolysin O (LLO) (22). Their extended specificity suggested that nanosponges might represent a broadly applicable adjunctive therapy for the treatment of infectious diseases caused by PFT-producing bacterial pathogens.

RBC-derived nanosponges represent ideal candidates for adjunctive detoxification therapies for the treatment of intraocular infections, given their broad spectrum of activity, lack of toxicity, and *in vivo* stability (18–22). Supplementation of antibiotics with nanosponges might prove beneficial for the treatment of endophthalmitis as well as prophylactically for intraocular surgeries and procedures that risk introducing bacteria into the eye. We published that rabbit nanosponges reduced E. faecalis cytolysin-mediated damage to the retina and improved retinal function even in the absence of antibiotic treatment (18). Rabbit nanosponges were also nontoxic to mouse eyes and did not incite an inflammatory response directed toward heterologous RBC membrane-associated proteins (18), establishing the feasibility of utilizing nanosponges to detoxify and treat E. faecalis intraocular infections. In the present study, we evaluated the effectiveness of human nanosponges to serve as adjunctive therapy for the treatment of intraocular cytolytic E. faecalis infection and to test the broader applicability of human nanosponges as an adjunctive therapy for S. pneumoniae, MSSA, MRSA, and B. cereus endophthalmitis. As previously described, the cytolysin is the only PFT and, in most strains, the only toxin produced by E. faecalis. Cytolysin enhances E. faecalis virulence in multiple animal infection models (40–42), including endophthalmitis (9, 10), and in epidemiological studies has been associated with patient mortality (43). As a factor that is enriched among virulent antibiotic-resistant isolates, the cytolysin represents an ideal candidate for evaluating the feasibility of antitoxin adjunct therapies. Direct injection of rabbit nanosponges alone into the vitreous 6 h postinfection with cytolytic E. faecalis resulted in significant protection of retinal architecture and increased retinal function retention (18). In the present study, human nanosponge treatment of eyes infected with a cytolytic E. faecalis resulted in protection and improved retinal function similar to that observed with rabbit nanosponges (18). The combination of human nanosponges with the fluoroquinolone gatifloxacin significantly reduced bacterial burdens but improved retinal function retention to a significantly higher degree than human nanosponges alone. The addition of gatifloxacin also reduced ocular pathological changes to a greater extent than human nanosponges alone. Importantly, human nanosponges did not interfere with the *in vivo* killing of E. faecalis by gatifloxacin, an important aspect for consideration in development of a combination antibiotic-antitoxin therapy. However, human nanosponges did not augment the activity of gatifloxacin alone. The cytolysin varies in activity depending on the target cell species (27, 28), which may be due to variations in phosphatidylcholine concentrations in the membrane outer leaflet (44). Human cells might be more sensitive than mouse cells to the cytotoxic effects of the cytolysin, and thus addition of human nanosponges to antibiotics for human infection might be more beneficial. In our previous study, intraocular infection with an isogenic noncytolytic E. faecalis strain resulted in 69.5% A-wave retention and 58.6% B-wave retention (18), levels resulting from the complete absence of the cytolysin. Treatment of a cytolytic E. faecalis infection with gatifloxacin alone, or gatifloxacin plus human nanosponges, resulted in only approximately 50% A- and B-wave retention in the present study, suggesting that nonneutralized cytolysin may still have been present in these eyes. Altering the timing of administration or increasing the concentration of human nanosponges might serve to better augment gatifloxacin in further reducing bacterial concentrations, decreasing toxin levels, and reducing retinal damage.

To explore the possibility of extending the use of human nanosponges as an adjunctive therapy for the other common Gram-positive intraocular pathogens, we further tested their capacity to deplete hemolytic activity from culture supernatants *in vitro* and their effectiveness at improving outcomes in conjunction with gatifloxacin in our live infection model. Since S. pneumoniae, like E. faecalis, produces a single PFT, we hypothesized that human nanosponges might be as effective in neutralizing pneumolysin. Human nanosponges effectively neutralized the S. pneumoniae pneumolysin and its hemolytic activity *in vitro* and significantly improved retinal function and reduced inflammation in a sterile endophthalmitis model. In the live infection model, human nanosponge treatment mitigated inflammation and retinal function loss either alone or in conjunction with gatifloxacin. However, no synergistic effect with gatifloxacin was observed. Sanders et al. demonstrated that immunization with a nontoxic pneumolysin variant elicited antibodies that reduced the destruction of ocular tissues (17). Antibiotics were not tested in that study. However, Ghate et al. found that following intraocular infection with S. pneumoniae, intravitreal injection of a combination of antipneumolysin antibody and vancomycin did not provide significant additional therapeutic benefits to that by vancomycin alone (45). These authors treated rabbit eyes with either antipneumolysin antibody and vancomycin or vancomycin alone 24 h following intravitreal infection with S. pneumoniae. Inclusion of the antipneumolysin antibody resulted in increased anterior chamber and vitreous inflammation relative to that in eyes treated with vancomycin alone (45). In contrast, we did not observe increased inflammation in eyes treated with human nanosponges plus gatifloxacin relative to that in eyes treated only with gatifloxacin, and reported that rabbit nanosponges did not incite a significant inflammatory response (18). Similar to our results, Ghate et al. observed no significant differences in retinal function response between eyes treated with antipneumolysin-vancomycin and eyes treated with vancomycin only (45). It is possible that any potential synergistic effect might be countered by immune effector cell-mediated bystander damage that antitoxin therapies and antibiotics do not directly arrest.

In contrast to human nanosponge activity against E. faecalis and S. pneumoniae, none of the treatment groups showed significantly improved retinal function following B. cereus infection. This contrasted with the *in vitro* hemolysis assays that showed significant neutralization of B. cereus hemolytic activity and the *in vivo* sterile endophthalmitis model that showed retinal function protection after pretreatment of B. cereus culture supernatants with rabbit nanosponges. Interestingly, we observed decreased ocular pathology and less cellular infiltrate in eyes infected with B. cereus following treatment with human nanosponges alone and with human nanosponges in combination with gatifloxacin. A continued decline in function might have resulted from B. cereus PFT saturation of the human nanosponges and/or the presence of non-PFTs. Upon entry into the vitreous, B. cereus replicates rapidly, produces a broad array of PFTs and non-PFTs, and incites a vigorous inflammatory response (6–8). Six hours postinfection was selected for treatment administration, as this represents a point when B. cereus concentrations reach approximately 10^6^ CFU/eye but retinal function retention remains relatively high (46, 47). PFT levels might have saturated the human nanosponges without the presence of gatifloxacin to halt bacterial replication and further toxin production. Non-PFT production and nonneutralized PFTs might have contributed to the continued decline in retinal function. Although the inclusion of gatifloxacin improved retinal function to some degree, this improvement was not statistically significant relative to that in untreated mouse eyes. Even though the addition of gatifloxacin failed to significantly arrest further functional decline, bacterial concentrations were reduced by approximately 6 orders of magnitude at 12 h postinfection. This decline in retinal function may have resulted from activities of other non-PFTs and/or retinal toxicity from neutrophils infiltrating into the eye beginning at approximately 4 h postinfection (47). A similar scenario may hold true for B. cereus*-*infected eyes treated with gatifloxacin only.

Both B. cereus and S. aureus present a more challenging therapeutic situation than E. faecalis and S. pneumoniae, as B. cereus and S. aureus produce both PFTs and non-PFTs. Hu et al. demonstrated that subcutaneous injection of a mixture of nanosponges with purified S. aureus alpha-toxin prevented significant tissue damage and inflammation (19). This suggests that similar results might be achieved following intraocular injection of nanosponge-treated S. aureus supernatants. In the present study, rabbit nanosponges significantly neutralized both MSSA and MRSA hemolytic activity *in vitro*, but rabbit nanosponges neutralized MSSA hemolytic activity to a greater extent (93% reduction) than it neutralized the hemolytic activity of the MRSA isolate (66% reduction). This difference in neutralization activity was reflected in the sterile endophthalmitis model. Rabbit nanosponge pretreatment of S. aureus supernatant resulted in significantly higher retinal function retention, but pretreatment of MRSA supernatant did not result in significant improvement. Both strains exhibited virtually identical hemolytic activity *in vitro* (82.1% for the MSSA strain versus 82.7% for the MRSA isolate), suggesting that the rabbit nanosponges possessed a lower capacity for neutralizing alpha-toxin and/or the other PFTs produced by the MRSA isolate. Alternatively, differences in the relative expression of alpha-toxin, a pore-forming hemolysin, and beta-toxin, a hemolytic sphingomyelinase (29, 30), between the two strains might account for this observation. Differences in the relative production of the non-pore forming γ- and δ-hemolysins might have also played a role in the reduced capacity of rabbit nanosponges to neutralize MRSA supernatant. In the live infection model, however, human nanosponge treatment either alone or in conjunction with gatifloxacin did not significantly afford retinal protection from infection with the methicillin-sensitive S. aureus strain. Gatifloxacin treatment alone also did not result in improved retinal function. Surprisingly, gatifloxacin had no effect on bacterial concentrations in these eyes. This was unexpected given that the MSSA strain has not been reported to be resistant to gatifloxacin (48). Gatifloxacin-treated groups showed a decrease in inflammation, whereas nanosponge-treated eyes appeared identical to untreated eyes. These results suggested that human nanosponges were not able to prevent retinal function decline regardless of the presence of gatifloxacin. This may have been the result of the continued growth and production of PFTs that saturated the human nanosponges and overwhelmed neutralization capacity, as well as the production of non-PFTs. However, even though human nanosponges and gatifloxacin alone did not significantly mitigate retinal function decline after intraocular MRSA infection, human nanosponges augmented gatifloxacin in reducing the bacterial load and increasing retinal function. This improved effect relative to that in the MSSA strain may have been due to increased killing of the MRSA isolate by gatifloxacin, preventing nanosponge saturation. These results support the prospect of using human nanosponges as an adjunctive therapy to antibiotics as a treatment for MRSA endophthalmitis. This is of clinical relevance, given that the frequency of MRSA endophthalmitis cases is on the rise (31–33).

In summary, rabbit nanosponges effectively reduced the hemolytic activity of a diverse array of PFTs produced by the common Gram-positive endophthalmitis pathogens and protected retinal function in a sterile endophthalmitis model. Human nanosponges alone protected mouse retinas following intraocular infection with E. faecalis and S. pneumoniae but not after B. cereus or MSSA infection. In conjunction with gatifloxacin, nanosponges significantly improved retinal function retention over gatifloxacin alone following infection with MRSA. These results support the potential for biomimetic RBC-derived nanosponges to serve as a novel adjunctive therapeutic agent for the treatment of intraocular infections. Given that endophthalmitis is commonly caused by Gram-positive bacteria that elaborate one or more PFTs, and MRSA and multidrug-resistant strains of bacteria are becoming increasingly more common, novel therapies that address both bacterial replication and toxin production are urgently needed. Future studies to evaluate the pharmacokinetics of RBC-derived nanosponges in conjunction with antibiotics in the eye, as well as refinements to concentrations and timing of administration, will lay the foundation for the use of nanosponges as a new adjunct therapy for bacterial intraocular infections.

## MATERIALS AND METHODS

### Bacterial strains and nanosponges.

The following bacterial strains were used in these studies: Bacillus cereus ATCC 14579, Enterococcus faecalis FA2-2 (pAM714) (wild-type cytolysin producer), methicillin-resistant Staphylococcus aureus ocular isolate 180 (MRSA), laboratory and methicillin-sensitive S. aureus strain 8325-4 (MSSA), and Streptococcus pneumoniae TIGR4. All of these strains have been used to initiate endophthalmitis in mice and rabbits (6–10, 18, 49, 50). E. faecalis, MSSA, MRSA, and B. cereus were grown in brain heart infusion (BHI) medium at 37°C for 18 h. S. pneumoniae was cultivated in Todd Hewitt broth (THB) supplemented with 0.5% yeast at 37°C with 5% CO_2_ for 18 h. For the *in vitro* hemolysis assays and sterile endophthalmitis model, S. pneumoniae, MSSA, MRSA, and B. cereus sterile culture supernatants were prepared by centrifugation of the cultures for 10 min at 4,300 × *g*, and the supernatants were passed through a 0.22-μm Millex GP filter unit (Merck Millipore Ltd., Tullagreen, Ireland). Supernatants were maintained on ice prior to all assays. For the live infection endophthalmitis model, E. faecalis, S. pneumoniae, MSSA, MRSA, and B. cereus were grown as described above. E. faecalis, S. pneumoniae, and B. cereus were diluted to 100 CFU per 0.5 μl, and MSSA and MRSA were diluted to 5,000 CFU per 0.5 μl. Rabbit nanosponges were prepared as previously described (18). Polymeric cores were generated by adding 10 ml of poly(lactic-co-glycolic acid) (PLGA) (carboxyl acid-terminated, 0.67 dl/g, 50:50 monomer ratio; LACTEL absorbable polymers) polymer (20 mg/ml in acetone) to 20 ml of Tris-HCl buffer (10 mM, pH 8). The solution was stirred and allowed to evaporate for 2 h. For membrane coating, purified rabbit RBC membranes were first mixed with PLGA cores at a protein-to-polymer weight ratio of 1:4, followed by sonication in a Fisher FS30D bath sonicator for 10 min. Size and zeta potential of the RB nanosponges were measured by dynamic light scattering using a Malvern ZEN 3600 Zetasizer (19). Rabbit nanosponges were approximately 90 nm in diameter and possessed a surface zeta potential of −35 mV. Human nanosponges were prepared in a similar manner. Packed human red blood cells (hRBCs) from healthy donors were purchased from ZenBio, Inc. Packed hRBCs were washed with ice-cold 1× PBS and then suspended in hypotonic 0.25× PBS in an ice bath for 20 min for hemolysis. Lysed cells were centrifuged at 800 × *g* for 5 min, followed by hemoglobin removal. The hypotonic treatment was repeated three times, and purified membranes were collected. Polymeric cores made from poly(lactic-co-glycolic acid) (PLGA) were prepared with a nanoprecipitation method, where 1 ml of PLGA (20 mg/ml in acetone) was added dropwise into 3 ml of water. The mixture was stirred for 2 h for the organic solvent to evaporate. Nanosponges were then prepared by mixing hRBC membranes with PLGA cores, followed by bath sonication for 10 min.

### *In vitro* hemolysis assays.

Bacterial sterile supernatants were mixed with equal volumes of 8 mg/ml rabbit nanosponges or phosphate-buffered saline (PBS) and incubated for 30 min. For each strain, maximum neutralization of their respective toxins was reached within 30 min of incubation with rabbit nanosponges. The rabbit nanosponges were then removed via centrifugation at 2,900 × *g* for 5 min. Hemolytic assays were performed on treated or untreated supernatants by adding washed rabbit erythrocytes to a final volume at 5% and incubating for 30 min at 37°C. Unlysed erythrocytes were removed by centrifugation at 500 × *g* for 5 min. Hemoglobin release was quantified by measuring the optical density at a wavelength of 490 nm using a FLUOstar Omega microplate spectrophotometer (BMG Labtech, Inc., Cary, NC). Values are expressed as percentage hemolysis relative to a 100% lysis control in which 5% rabbit erythrocytes were lysed in double-distilled water (ddH_2_O). Values represent the means ± the standard deviations from three independent experiments.

### Murine sterile and live infection endophthalmitis models.

This study was carried out in strict accordance with the recommendations in the Guide for the Care and Use of Laboratory Animals of the National Institutes of Health. The protocol was approved by the Institutional Animal Care and Use Committee of the University of Oklahoma Health Sciences Center (protocol numbers 15-103 and 18-079). Six-week-old C57BL/6J mice were acquired from the Jackson Laboratory (catalog 000664; Bar Harbor ME). Mice were acclimated to conventional housing 1 week prior to injection to allow for physiological and nutritional stabilization and to equilibrate their microbiota. All mice were housed under microisolation conditions on a 12-h on/12-h off light cycle prior to the experiments and then under biosafety level 2 conditions during experiments. Mice were 8 to 10 weeks of age at the time of the experiments.

Mice were anesthetized with a combination of ketamine (85 mg/kg body weight) (Ketathesia; Henry Schein Animal Health, Dublin, OH) and xylazine (14 mg/kg body weight) (AnaSed; Akorn Inc., Decatur, IL). Intravitreal injections were performed with sterile borosilicate glass micropipettes (Kimble Glass Inc., Vineland, NJ, USA) beveled to an approximate bore size of 10 to 20 μm (BV-10 KT Brown type micropipette beveller; Sutter Instrument Co., Novato, CA, USA). Eyes were visualized with a stereomicroscope, and the micropipettes were inserted just posterior to the superior limbus. For the sterile endophthalmitis model, right eyes were injected with 0.5 μl of bacterial sterile supernatants not treated or treated with rabbit nanosponges directly into the midvitreous. Left eyes served as uninjected controls. For the live infection endophthalmitis model, 100 CFU of either E. faecalis, S. pneumoniae, or B. cereus in 0.5 μl was injected into the right eyes of mice. For MSSA and MRSA, 5,000 CFU in 0.5 μl was injected. Left eyes served as uninfected controls. Mice were allowed to recover for 6 h, at which time they were anesthetized with ketamine and xylazine as described above and were intravitreally injected with 0.5 μl PBS only (untreated control), 0.5 μl PBS containing 1.25 μg gatifloxacin (Zymaxid 0.5%; Allergan, Inc., Irvine, CA), 0.5 μl PBS containing 1.25 μg gatifloxacin and 2 μg of human nanosponges, or 0.5 μl PBS containing 2 μg of human nanosponges. Injection rates and volumes were monitored using a programmable cell microinjector (Microdata Instruments, Plainfield, NJ, USA).

### Scotopic electroretinography.

For the sterile endophthalmitis model, mice injected with supernatants either treated or not treated with rabbit nanosponges were dark adapted for 24 h prior to ERGs. For the live infection model, mice infected with B. cereus were dark adapted for 12 h, and mice infected with E. faecalis, S. pneumoniae, MSSA, and MRSA were dark adapted for 24 h prior to ERGs. All mice were anesthetized as described above, and their eyes dilated with topical phenylephrine. Topical anesthetic (0.5% proparacaine HCl) was applied to eyes prior to conducting the ERGs. Gold-wire electrodes were placed on the cornea of each eye, and reference electrodes were attached to the head and tail of the mouse. Five white-light flashes were administered consecutively to the mouse 60 s apart (10-ms duration) in order to provoke a retinal response. Scotopic A-wave (corresponding to photoreceptor cell activity) and B-wave (corresponding to Müller, bipolar, and amacrine cell activity) amplitudes were recorded for each eye (Espion E2; Diagnosys, LLC, Lowell, MA). Immediately following ERG, mice were euthanized by CO_2_ asphyxiation prior to harvesting the eyes for thin-section histology or bacterial quantification. The percentage of retinal function retained in the infected eye was calculated in comparison with uninfected left eye controls as 100 – {[1 – (experimental A- or B-wave amplitude/control A- or B-wave amplitude)] × 100}. Values represent the means ± the standard deviations from a sample size of 6. Two independent experiments were performed.

### Thin-section histology.

For the sterile endophthalmitis model, eyes were harvested 24 h after injection of 0.5 μl of sterile supernatants treated or not treated with rabbit nanosponges. For the live infection model, eyes were harvested after 12 h from B. cereus-infected mice, and 24 h from E. faecalis-, S. pneumoniae-, MSSA-, and MRSA-infected mice. Harvested eyes were incubated in High Alcoholic Prefer fixative for 2 h at room temperature. Eyes were then transferred to 70% ethanol, embedded in paraffin, sectioned, and stained with hematoxylin and eosin. Images are representative of at least 3 eyes from 2 independent experiments.

### Bacterial quantitation.

Eyes were removed, placed into separate tubes containing 400 μl of sterile PBS and 1.0-mm sterile glass beads (Biospec Products Inc., Bartlesville, OK), and homogenized for 60 s at 5,000 rpm in a Mini-BeadBeater (Biospec Products, Inc., Bartlesville, OK). Eye homogenates were serially diluted and plated in triplicates on BHI agar plates for E. faecalis, MSSA, MRSA, and B. cereus, or THB plus 0.5% yeast agar plates for S. pneumoniae. E. faecalis, MSSA, MRSA, and B. cereus plates were incubated at 37°C, and S. pneumoniae plates were incubated at 37°C with 5% CO_2_. After overnight incubation, the CFU per eye was determined as previously described (18, 46, 47). Values represent means ± standard deviations from at least 6 eyes per group in two independent experiments. For mice in the B. cereus- and S. pneumoniae-infected and gatifloxacin-treated groups whose eyes were sterilized (0 CFU/eye), the 0 was assigned the value of 1 to allow these data points to be plotted on a logarithmic scale.

### Statistics.

Data are the arithmetic means ± the standard deviations from all samples in the same experimental group in replicate experiments. Comparative differences between groups were taken to be statistically significant when the *P* value was <0.05. The Mann-Whitney U test was used to compare experimental groups for the *in vitro* hemolysis assays, ERG experiments, and bacterial counts per eye. All statistical analyses were performed using GraphPad Prism 6.05 (GraphPad Software, Inc., La Jolla, CA).
